# The Pathogenesis of the Neurofibroma-to-Sarcoma Transition in Neurofibromatosis Type I: From Molecular Profiles to Diagnostic Applications

**DOI:** 10.3390/cancers17243955

**Published:** 2025-12-11

**Authors:** Sabrina Busciglio, Ilenia Rita Cannizzaro, Anita Luberto, Antonietta Taiani, Barbara Moschella, Enrico Ambrosini, Sofia Cesarini, Mirko Treccani, Cinzia Azzoni, Lorena Bottarelli, Domenico Corradi, Vera Uliana, Davide Martorana, Valeria Barili, Antonio Percesepe

**Affiliations:** 1 Medical Genetics, Department of Medicine and Surgery, University of Parma, 43126 Parma, Italy; 2 Medical Genetics, University Hospital of Parma, 43126 Parma, Italy; 3 Department of Food and Drug, University of Parma, 43126 Parma, Italy; 4 Pathology Unit, Department of Medicine and Surgery, University of Parma, 43126 Parma, Italy

**Keywords:** Neurofibromatosis type 1, malignant peripheral nerve sheath tumors, liquid biopsy, genomic profiling, epigenetic dysregulation, omics analysis

## Abstract

Neurofibromatosis type 1 (NF1) predisposes to a spectrum of peripheral nerve sheath tumors, ranging from benign plexiform neurofibromas (PN) to atypical neurofibromatous neoplasms of uncertain biological potential (ANNUBP) and malignant peripheral nerve sheath tumors (MPNST). Tumorigenesis follows a multistep molecular cascade initiated by biallelic *NF1* inactivation, followed by *CDKN2A* loss and disruption of the Polycomb Repressive Complex 2 (PRC2). These events guide chromatin remodeling, widespread epigenetic dysregulation, and activation of oncogenic pathways such as RAS/MAPK and PI3K/AKT. Here, we integrate genomic, transcriptomic, and epigenomic studies to delineate the molecular trajectories underlying tumor progression and to define promising biomarkers for the early detection of malignant transformation. Emerging liquid biopsy approaches, based on circulating tumor DNA (ctDNA) analyses, reveal distinctive copy number variations (CNVs) and methylation patterns that mirror tissue-derived profiles, enabling the detection of malignant transformation. Together, these findings support a model in which cumulative genetic and epigenetic alterations drive the PN–ANNUBP–MPNST continuum. They also underscore the value of multi-omics and liquid biopsy-based strategies to improve early diagnosis, patient risk stratification, and personalized management of NF1-associated tumors, thereby advancing precision medicine in this complex disease spectrum.

## Simple Summary

Patients with Neurofibromatosis type 1 can develop different types of nerve sheath tumors, though only a small fraction become malignant. Distinguishing benign plexiform neurofibromas from early signs of malignant transformation remains challenging using imaging or clinical symptoms alone. This review examines the molecular evolution of these tumors, describing the key genetic and epigenetic changes that drive the transition from benign lesions to malignant peripheral nerve sheath tumors (MPNST). By integrating evidence from multiple “omics” studies, we highlight the biological hallmarks of each stage of progression and explore emerging biomarkers that may help to identify patients at higher risk. We also discuss new liquid biopsy approaches, which analyze tumor-derived DNA fragments circulating in the blood and may allow earlier detection of malignant transformation. Understanding these mechanisms can improve surveillance strategies and support more personalized care for patientswith NF1.

## 1. Introduction

Neurofibromatosis type 1 (NF1) is a common autosomal dominant genetic disorder, affecting approximately 1 in 2500–3000 individuals worldwide. It is caused by pathogenic variants in the *NF1* gene located on chromosome 17q11.2 [1,2].

Diagnostic hallmarks include benign neurofibromas, which can be cutaneous, subcutaneous, or plexiform (PN), carrying a significant risk of malignant transformation [3]. Among the most life-threatening complications is the development of malignant peripheral nerve sheath tumors (MPNSTs), which occur in approximately 8–13% of individuals with NF1 and represent a major cause of NF1-related mortality [4].

The tumor progression of PN leads to the MPNST development, which is a soft-tissue sarcoma characterized by high recurrence (30–70%) after resection and resistance to chemotherapy [5]. Beyond neural manifestations, NF1 is a multisystem disorder involving skeletal, cardiovascular, and endocrine systems and is associated with an increased risk of other malignancies [6]. Thus, the NF1 clinical spectrum encompasses benign, intermediate, and malignant lesions, reflecting a multistep neoplastic process driven by sequential genetic and epigenetic events.

## 2. Molecular Pathogenesis and Genetic Drivers of NF1-Related Tumors

### 2.1. NF1 Loss and the Initiation of Plexiform Neurofibroma (PN)

Approximately half of individuals with NF1 develop PNs, which are benign, often congenital nerve sheath tumors. These tumors arise from multiple deep nerve fascicles and their branches and are characterized by a tortuous, infiltrative growth pattern that extends along the length of a nerve and into surrounding tissues. Histologically, PNs display distinctive features, such as coarse collagen bundles interspersed with loosely arranged, haphazard spindle cells with ill-defined cytoplasmic borders and an absence of degeneration or necrosis, that clearly differentiate them from MPNST. Mitotic figures are rare, usually fewer than 1 per 50 high-power fields (HPF which corresponds to 0.2 mm^2^). The tumor is composed predominantly of wavy Schwann cells (S100-positive), interlaced with a lattice-like network of fibroblasts (CD34-positive) and scattered degranulated mast cells. The nuclei of neurofibroma cells are characteristically comma-shaped, and only a small fraction (<2–5%) of cells show Ki-67 immunoreactivity, reflecting the low proliferative index of these lesions [7].

Bi-allelic inactivation in the *NF1* gene is the initiating event in neurofibroma formation [8,9,10] and a crucial precursor to malignancy, especially in NF1-associated MPNSTs [11,12]. The *NF1* gene encodes neurofibromin, a protein that acts as a negative regulator of RAS (Rat sarcoma virus) signaling. Neurofibromin contains a GTPase-activating protein (GAP)-related domain of approximately 300 amino acids, which stimulates the intrinsic GTPase activity of RAS, accelerating the conversion of active RAS-GTP to its inactive RAS-GDP state. Given the central role of RAS in cell cycle regulation and tissue growth, most studies have focused on its RAS-GAP function. Through this mechanism, neurofibromin negatively impacts several downstream proteins, including RAS–MEK–ERK (MAPK), RAS–AKT–mTOR, and RAS–Rac1 pathways [13].

Although individuals with NF1 inherit a germline variant in one *NF1* allele, a single mutated copy is insufficient for tumor formation. Rather, loss-of-function (LOF) mutations and complete absence of neurofibromin expression are necessary for the development of benign and malignant tumors [13]. This leads to continuous RAS signaling, which promotes cell proliferation via the MAPK cascade, and enhances cell survival by inhibiting apoptosis through the PI3K/AKT/mTOR pathway. Somatic loss of heterozygosity (LOH), for instance, represents a common mechanism of LOF of the remaining wild type allele in somatic cells such as Schwann cells, which give rise to neurofibromas [14,15]. LOH may arise in somatic cells through a number of mechanisms, including deletions, whole chromosome loss due to nondisjunction with or without re-duplication. However, mitotic recombination has been shown to be the most frequent common event accounting for LOH in NF1-associated tumors [15].

The constitutive RAS hyperactivation associated with biallelic *NF1* inactivation has been documented in both human and murine NF1-associated tumors [13]. The best-characterized downstream effector is the MAPK pathway: increased RAS-GTP activates RAF kinase, which sequentially phosphorylates MEK and ERK1/2 kinases, resulting in transcriptional activation of genes that promote cell proliferation and differentiation [16]. Dysregulation of this pathway is observed in the majority of MPNSTs, and BRAF mutations or amplifications, which can also activate ERK signaling, are particularly common in sporadic MPNST [17].

Another major RAS effector branch is the PI3K/AKT/mTOR pathway. Neurofibromin loss enhances PI3K and AKT activity, leading to phosphorylation and inactivation of the tuberin (TSC2) protein and consequent constitutive mTOR activation [16]. This axis supports cell survival, growth, and metabolism. Both mTORC1 and mTORC2 contribute to NF1 tumorigenesis, and pathway hyperactivation is consistently detected in NF1-deficient cells and tumors.

Together, these findings define *NF1* loss as the initiating molecular event that drives RAS pathway hyperactivation, laying the basis for benign PN formation and subsequent progression toward malignant transformation in NF1-associated tumors.

### 2.2. Key Cooperating Alterations in Progression to ANNUBP and Malignancy to MPNST

Patients with NF1-associated MPNST have lower survival rates and greater resistance to therapy compared to those with sporadic MPNST. However, complete resection of premalignant lesions can significantly reduce mortality and recurrence, highlighting the importance of early diagnosis and surgical excision of precursor lesions before tumors progress to high-grade or metastatic disease. There are dramatic histological alterations that distinguish PN from MPNST. Indeed, MPNSTs exhibit striking cytologic atypia, with cells showing nuclear enlargement, pleomorphism, and loss of characteristic neurofibromatous architecture. Instead, they form intersecting fascicles arranged in herringbone or storiform patterns. Mitotic activity is markedly increased (>10 mitoses per 10 HPF). While some regions may show more uniform, spindle-shaped cells, areas of perivascular hypercellularity and focal necrosis are common [18].

Interestingly, an intermediate stage between PN and MPNST, now classified as an atypical neurofibromatous neoplasm of uncertain biological potential (ANNUBP), may represent a pre-malignant step in the pathological continuum of malignant transformation [19]. ANNUBPs feature hyperchromatic nuclei, increased cellularity, and disruption of the typical neurofibroma architecture, sometimes with herringbone or storiform fascicular growth pattern, but lack necrosis. The ANNUBPs are histologically intermediate lesions that display increased cellularity, nuclear atypia, and low mitotic activity (>1 per 50 HPF but <3 per 10 HPF), without infiltrative growth. They often arise within pre-existing PNs and may show heterogeneous or reduced expression of Schwann-cell lineage markers (S100, SOX10), as well as loss of p16 immunoreactivity indicative of *CDKN2A* (Cyclin-dependent kinase inhibitor 2A) deletion or inactivation. These features support their classification as lesions at risk for malignant progression rather than overt sarcoma [20].

At the molecular level, the histological emergence of ANNUBPs marks a transition toward genomic instability, with recurrent *CDKN2A* (9p21) deletions frequently appearing early, often preceding clear definitive malignancy [21,22].

In contrast, high-grade MPNSTs display profound chromosomal instability, genome-wide CNVs, aneuploidy, and epigenetic deregulation [23,24,25]. Moreover, several studies in MPNST showed the inactivation of other additional tumor suppressor genes such as *RB1* (Retinoblastoma 1), *TP53* (Tumor protein p53), and *PTEN* (Phosphatase and Tensin homolog) in both NF1-associated and sporadic MPNSTs, highlighting a driving role of mutations in these genes in tumor malignancy [23].

Additionally, in malignant progression additional cooperating alterations amplify tumorigenic signaling such as the loss in the Polycomb Repressive Complex 2 (PRC2) which results in global depletion of the repressive histone mark H3K27me3 (trimethylation of lysine 27 on histone H3), enhancer reprogramming, and dedifferentiation of Schwann cells into invasive, therapy-resistant states, promoting malignant transformation [26].

**Figure 1:**
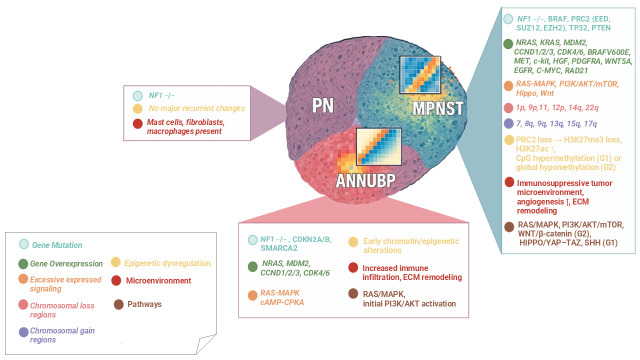
Molecular transition of PN–ANNUBP–MPNST in NF1-related tumors.

From a diagnostic standpoint, taken together biallelic *NF1* loss and *CDKN2A/B* define the ANNUBP state, while PRC2 loss (±*TP53*) with widespread CNVs and other pathway rewiring (RTKs, WNT/HIPPO, and PI3K–mTOR) marks the malignant conversion to MPNST. Overall, this progression represents a stepwise molecular continuum driven by cumulative genetic, epigenetic, and microenvironmental alterations. Figure 1 schematically summarizes these events, illustrating the transition from PN to ANNUBP and finally to MPNST, along with the predominant pathways and cellular mechanisms involved. Regardless, there must be multiple genomic alterations before neurofibromas transform into malignant tumors; how molecular changes may drive the process of tumor deterioration is not fully clarified.

Stepwise model of tumor progression in NF1 associated peripheral nerve sheath tumors (PNSTs), showing the transition from plexiform neurofibroma (PN) to atypical neurofibromatous neoplasm of uncertain biological potential (ANNUBP) and malignant peripheral nerve sheath tumor (MPNST). Each stage displays progressive alterations. Color-coded boxes summarize the predominant alterations observed at each stage.

Symbol explanation:

The horizontal connector (→) indicates a sequential progression from one molecular alteration to the next within the same stage.

The upward arrow (↑) indicates an increased level or upregulation of the associated biological process or signaling pathway.

## 3. Omics Insights in NF1-Associated Tumors

### 3.1. Genomic Landscape and Evolution to MPNST

Whole exome sequencing (WES) and whole genome sequencing (WGS) have significantly advanced the understanding of cancer genomics and have been widely used to identify the characteristic mutational patterns of MPNSTs. Although the first studies on MPNST genomic landscape have been conducted in relatively small patients’ cohorts, they allowed the detection of recurrent molecular events common to MPNSTs, without identifying uniform molecular markers for all tumors belonging to this specific histological group [27]. This gap largely stems from the low incidence of MPNST, which limits the availability of sufficient tumor samples for detailed investigation. These challenges led to the creation of the international consortiums such as the Genomics of MPNST (GeM), that aims at performing multi-omic analysis on MPNST samples to better clarify the time sequence and nature of genetic events associated with cancer onset and progression [27].

Although no recurrent genetic alterations have been detected in benign neurofibromas, except for the *NF1* gene variants, MPNST displays a diverse mutational spectrum including multiple gene mutations and DNA fragment deletions or duplications. As mentioned above other than *NF1* biallelic mutations, several other tumor suppressor genes have been detected as altered in MPNST tumors also in sporadic form, such as *CDKN2A*, *RB1*, *TP53*, and *PTEN*. Early inactivation of both *CDKN2A* copies appears to be a necessary condition for the formation of ANNUBP, as initially demonstrated by Beert et al. and later confirmed by Carrió et al. [28,29,30,31].

Several studies have shown that *RB1* loss is a central event driving uncontrolled Schwann cell proliferation and MPNST development. Under physiological conditions, RB1 restrains the G1/S transition by binding and inhibiting E2F transcription factors, thereby enforcing cell-cycle arrest and senescence. In Schwann cells, this RB1-dependent checkpoint is particularly important, as it prevents hyperproliferation of PNs and acts as a barrier to malignant progression [32,33].

In MPNSTs, the RB1 inactivation removes this checkpoint entirely, allowing continuous E2F activity, uncontrolled cell-cycle entry, and tumor growth. Importantly, the oncogenic driver *RABL6A*, which is highly expressed in MPNSTs, promotes tumorigenesis precisely by antagonizing the RB1 pathway, accelerating RB1 inactivation and thereby enabling persistent proliferation [32,33].

Moreover, the status of RB1 critically influences the efficacy of CDK4/6 inhibitors. These inhibitors function by preventing RB1 phosphorylation, thus keeping RB1 in its active, growth-suppressive form. Consequently, their efficacy depends on the presence of functional RB1. Tumors with complete RB1 loss are typically resistant to CDK4/6 blockade. However, in MPNSTs driven by RABL6A-mediated RB1 suppression, RB1 is not completely deleted; instead, its pathway is inhibited but not absent, which creates a therapeutic vulnerability. In this context, CDK4/6 inhibitors can restore RB1 activity, re-establishing cell-cycle arrest and reducing tumor growth, as demonstrated in RB1-dependent MPNST models [32].

Other alterations have been detected in the PTEN protein, a phosphatase responsible for inhibition of the PI3K/AKT signaling pathway. Indeed, *PTEN* mutations or loss led to hyperactivation of the PI3K/AKT pathway, promoting the survival and proliferation of MPNST cells [34]. Additionally, *PTEN* downregulation has been detected through promoter methylation to further reinforce PI3K/AKT/mTOR signaling [35].

Regardless of the mutational heterogeneity within these malignant tumors, inactivating alterations in the *TP53* gene are frequent changes detected in MPNST (up to 25%), which result in a loss of DNA damage response, increasing genomic instability and promoting tumor aggressiveness [36]. The *TP53* gene encodes the p53 protein, central tumor suppressor responsible for the surveillance of DNA damage and for promoting apoptosis. Peacock and colleagues analyzed longitudinal genomic evolution in clinical cases of NF1-associated MPNST, identifying early hemizygous microdeletions in the *NF1* and *TP53* genes. Furthermore, a nonsynonymous coding mutation in the *TP53* gene, present in the context of hemizygosity, was found. This suggests a mechanism where a functional alteration of *TP53* is accompanied by the loss of the normal allele, contributing to tumor progression through inactivation of the entire p53 pathway [37]. These recurrent genetic events suggest a fundamental role of these genes in the pathogenesis of MPNST. However, the non-universality of such alterations among all cases indicates that, despite being highly relevant from a biological perspective, they do not represent a unique genetic signature for a more accurate and generalizable molecular classification of MPNSTs.

Additional drivers accumulate, including receptor tyrosine kinase (RTK) amplifications, which involve key signaling receptors such as MET/HGF, EGFR, PDGFRA, and IGF1R [23,38], leading to constitutive activation of downstream proliferative and survival pathways and promoting aggressive growth, invasion, and therapeutic resistance. In NF1-associated MPNSTs, RTK activation often coexists with the loss of canonical tumor suppressors (such as *NF1*, *TP53*, and *CDKN2A*), generating a synergistic effect that drives cell-autonomous mitogenic signaling and enhances tumor progression. Importantly, these recurrent RTK amplification events may be fundamental to their potential as therapeutic targets, with ongoing efforts exploring multi-kinase inhibitors and RTK-directed combination therapies to overcome pathway redundancy and resistance mechanisms [39,40,41].

*BRAF* activating mutations or amplifications, such as *BRAF V600E* and *NRAS Q61* mutations, have been detected specifically in sporadic MPNST [42]. Less frequent changes, such as *AURKA* gain, *TYK2* activation, and *ATRX* alterations, are linked to maintenance of the alternative lengthening of telomeres as another mechanism of tumor survival [17,23].

In a large subset of cases, a distinctive defining event is the inactivation of PRC2, most often caused by mutations or deletions in *EZH2* (Enhancer of Zeste homolog 2), *SUZ12* (Suppressor of Zeste 12 homolog) or *EED* (Embryonic ectoderm development). This results in loss of histone H3 lysine 27 trimethylation (H3K27me3), an epigenetic change that leads to strong gene deregulations and serves as a useful diagnostic marker [43]. Several studies clearly highlight the critical role of the PRC2 loss in driving MPNST development and metastatic progression, comprising increased invasion, upregulation of matrix-remodeling enzymes, and elevated lung metastasis [44]. PRC2 somatic alterations are found in 70% of NF1-associated, 90% of sporadic and radiotherapy-associated MPNSTs, underscoring the critical role of the epigenetic dysregulation in MPNST pathogenesis [26,43]. Although the loss of *EZH2*, *SUZ12* and *EED* emerges as one of the most recurrent events, the frequencies and combinations of these alterations vary significantly across cohorts and models. In fact, exome sequencing of a large set of MPNST lines derived from primary tumor xenografts reported *SUZ12* loss in 62.5% of the samples, while the *TP53* loss was present in only 12.5% [45]. Similarly, another study observed loss of H3K27me3 in 55% of MPNST, with biallelic inactivation of *SUZ12* and *EED* in 28% and 17% of cases, respectively [46].

Consistent with this escalation in genomic complexity, longitudinal and cross-sectional datasets demonstrate that widespread chromosomal gains and losses emerge only at the MPNST stage, whereas earlier lesions such as ANNUBP show limited copy number changes (primarily *CDKN2A* loss) [22,23].

In 2015, Hirbe et al. performed analyses of matched samples from different disease stages, ranging from PN to primary and metastatic MPNSTs, showing a branched evolutionary pattern in which new driver events accumulate during malignant progression [47]. While pathogenic SNVs are enriched in primary tumors, metastatic lesions exhibit increased CNVs, reflecting increased genomic instability at later stages [46,48,49,50]. Notably, alterations in canonical MPNST-associated genes such as *TP53* and *SUZ12* were often confined to primary tumors, underscoring distinct molecular trajectories between primary and metastatic disease. Collectively, these findings suggest that metastatic MPNSTs evolve through divergent genetic mechanisms that only partially overlap with those driving primary transformation. This hypothesis was further supported by the study conducted by Godec et al. [47], which analyzed two MPNST-affected patients with multiple metastatic lesions to elucidate the genetic events involved in clonal progression and tumor spread. In both cases, metastases shared a common clonal origin with the primary tumor, maintaining distinctive genetic traits throughout the disease course. This suggests that cells with metastatic potential are already present within the primary neoplasm, and that dissemination results from the selection of specific adaptive subclones rather than the de novo acquisition of metastasis-specific mutations. However, the absence of shared point mutations across all samples highlights a pronounced genomic heterogeneity among tumor sites. In contrast, recurrent alterations in the *TRIM* gene family encoding E3-ubiquitin ligases involved in protein degradation and cell migration, were detected more frequently in metastatic than in primary tumors. Moreover, aberrant activation of additional signaling cascades beyond RAS has been increasingly recognized in MPNST pathogenesis. In particular, dysregulation of the Hippo and WNT pathways contributes to tumor growth and progression. Activation of the Hippo pathway effectors YAP and TAZ, characterized by their nuclear accumulation, has been documented both in patient-derived MPNST samples and in preclinical models, suggesting a role in promoting proliferation, stemness, and resistance to apoptosis. Similarly, altered WNT signaling, encompassing both canonical β-catenin dependent activation and non-canonical, context-dependent signaling mediated by Wnt5a, has been observed, further emphasizing the molecular complexity and crosstalk between oncogenic networks in MPNSTs [17].

Although the model has been largely elucidated by numerous studies highlighting key events such as (i) biallelic inactivation of *NF1*, as a common prerequisite; (ii) involvement of *TP53*; (iii) loss of function of PRC2 complex by mutations/deletions of *SUZ12* and *EED*, associated with global loss of H3K27me3; and (iv) progressive genomic complexification with large somatic CNVs in advanced stages, the precise molecular mechanisms determining the transformation of the PN, which is histologically classified as a benign tumor, are still largely unknown in MPNST.

### 3.2. Mutational Signatures and DNA Repair-Related Processes in MPNST

The complex mutational landscape of the MPNST may be characterized using COSMIC mutational signatures analysis [51] defining characteristic patterns of single base substitutions (SBS) to reveal the contributions of defective DNA repair mechanisms and other oncogenic processes.

In the GeM study, the single-base mutational load in MPNST was overall low, with no dominant exogenous signature comparable to UV or tobacco exposure, supporting a model in which chromosomal instability, rather than point mutations, drives tumorigenesis [30]. However, focused genomic analyses have begun to reveal discrete mutational patterns associated with specific DNA repair defects. In a recent sequencing comparison of superficial MPNST, deep MPNST, and desmoplastic/spindle melanomas, McAfee et al. [52] identified distinct COSMIC mutational signatures across these tumor types. While melanomas displayed UV-related SBS7a/b signatures, both superficial and deep MPNSTs clustered according to base-excision repair (BER)-associated signatures, specifically known as SBS30 and SBS36.

These signatures are linked to oxidative DNA damage and impaired repair of 8-oxo-guanine lesions, commonly involving *MUTYH* or *OGG1* dysfunction [53]. The absence of UV-related signatures further supports a distinct etiology for MPNST and implicates endogenous oxidative stress [30] as a potential driver of Schwann cell transformation. According to the COSMIC database, SBS30 represents the canonical signature of defective BER associated with 8-oxo-dG processing, whereas SBS36 reflects a related pattern resulting from *MUTYH* deficiency [53]. Their presence in MPNST genomes suggests that sustained oxidative stress and inefficient repair of oxidative lesions may synergize with chromosomal instability to promote malignant progression.

Other mutational processes appear less prominent. Some MPNST tumors exhibit clock-like signatures (SBS1 and SBS5), which reflect the accumulation of spontaneous deamination [53] of 5-methylcytosine over time, a ubiquitous background process rather than an MPNST-specific mechanism. Evidence for homologous recombination deficiency (HRD)-associated signatures, such as SBS3, remains anecdotal [53] and has not been substantiated in larger MPNST cohorts. Notably, a study on malignant transformation of vestibular schwannomas [54] revealed extensive copy number alterations but no consistent radiation- or HRD-related mutational signatures, reinforcing the concept that copy number instability, rather than defective double-strand break repair, dominates the mutational landscape of nerve sheath tumors.

### 3.3. Copy Number Variation Analysis to Detect Chromosomal Aberrations

Genomic instability, characterized by large gains, losses, and chromosomal rearrangements, is the main feature of MPNST. These alterations not only fuel accelerated tumor growth and invasive capacity but also contribute to the acquisition of therapeutic resistance. Therefore, understanding the processes that generate and sustain genomic instability, as well as its consequences for neoplastic progression, constitutes a crucial area of research. Clarifying these dynamics could pave the way for new targeted therapeutic strategies and enhance the effectiveness of treatments already available [55].

In MPNSTs, chromosomal rearrangements result in recurrent patterns of losses and gains in specific genomic regions. The most frequent losses affect chromosomes 1p, 9p, 11q, 12p, 14q, 17q and 22q, while gains are mainly concentrated on chromosomal arms 7p, 8q, 9q, 13q, 15q and 17q [17,45,56,57]. Among these regions, CNVs targeting key genes such as *NF1*, *CDKN2A*, and *TP53* are those relevant [9].

Notably, duplication of the chromosomal region 8q has been associated with lower overall survival in soft tissue sarcomas [45]. This is probably related to the amplification and subsequent overexpression of two positive regulators of cell proliferation: the proto-oncogene *c-MYC* and the protein RAD21, which is involved in DNA double-strand break repair and chromatid cohesion during mitosis.

Co-loss of *NF1* and *SUZ12*, both located at 17q11.2, occurs frequently, particularly in *NF1* microdeleted tumors and correlates with loss of H3K27me3 expression. Likewise, *EED* alterations contribute to PRC2 inactivation and epigenetic deregulation in MPNST. *TP53* loss or deletion at 17p13.1 is present in a subset of tumors, consistent with its involvement in late-stage genomic instability events [58].

Among recurrent gains, broad 8q amplification emerges as a near-universal clonal CNV associated with poor overall survival in soft-tissue sarcomas and MPNSTs. Candidate effectors include *MYC* (8q24), *RAD21* (8q24.11), and *HEY1*, all of which show increased expression in MPNST compared with PN [45].

Additional receptor tyrosine kinase loci are affected by 7p/7q gains, notably *EGFR* (7p11.2) and *MET* (7q31)/*HGF* (7q21), which sustain mitogenic signaling and tumor aggressiveness [41].

Finally, LOH involving 5q, 11q, 7p, and 22q, as well as amplifications of 2q and 9q, have been correlated with adverse clinical outcomes in the GeM multi-omics cohort, reinforcing the prognostic relevance of CNV profiling in MPNST [9].

However, reported CNV profiles in MPNSTs remain heterogeneous and sometimes controversial. For instance, Szymanski et al. described gains in chromosomes 1q, 7p, 8q, 9q and 17q [59], while Suppiah et al. observed amplifications in chromosomes 13q, 17p and 18q associated with poor prognosis, but not in 7p [60]. In a study conducted by Cortes-Ciriano et al., LOH of chromosome 5q was also closely associated with reduced overall survival, followed by LOH of chromosomes 11q, 7p and 22q and amplifications of 2q and 9q [9]. Recently, a strong association between distinct CNV profiles and H3K27me3 methylation status has been detected, which remains under investigation [9].

Overall, a unique aberrant chromosomal pattern characterizing all MPNSTs has not yet been identified. Discrepancies between studies may reflect differences in the investigated cohorts (like NF1-associated vs. sporadic individuals), tumor grade, or the genomic platforms used.

### 3.4. Transcriptomics and Single-Cell Studies

Few recent studies have provided insights into the early transcriptional programs characterizing benign and malignant lesions [61].

Comprehensive gene expression and pathway analyses have revealed stage-specific dysregulation of both tumor-intrinsic programs and the surrounding microenvironment during MPNST development. A recent study applied spatial transcriptomics in distinct regions within individual tumor samples. The authors demonstrated that some of the molecular events driving the transition from PN to MPNST may arise before such alterations become histopathologically evident. By performing multi-regional and longitudinal profiling of human PN-MPNST samples (n = 35), they identified three molecularly distinct subclusters representing different stages in the PN–MPNST continuum. Cluster 1, predominantly composed of PNs, showed upregulation of *CDKN2A*, which was significantly reduced in both Cluster 2 (ANNUBP-predominant) and Cluster 3 (MPNST-predominant) [61]. Cluster 2 showed strong enrichment of pathways associated with immune system processes and antigen presentation (including *HLA-DQA1*, *HLA-DQB1*, and *HLA-DRB1*), indicating an “immune-active” microenvironment. Concurrently, pathways related to epigenetic regulation, interferon signaling, and hypoxia were also enriched, suggesting the onset of functional immunosuppression. Interestingly, previous studies have shown that hypoxia in the tumor microenvironment can drive epigenetic dysregulation, impair immune cell cytotoxicity, and reduce interferon-gamma signaling [62].

These findings support a potential role for impaired antitumor immune responses in PN evolution and transformation. In contrast, with ANNUBPs, MPNSTs displayed further disruptions in apoptotic and angiogenic pathways, as well as in genes involved in mitotic regulation and cell cycle integrity, including *MAPK*, *PI3K/AKT*, *Notch*, *WNT*, *TGF-β*, and *JAK/STAT*. ANNUBPs were also characterized by gene signatures associated with enhanced immune surveillance and T-cell infiltration, both of which were markedly diminished in MPNSTs. Finally, ANNUBPs exhibited heterogeneous distribution across all subclusters [61].

Spatial intratumoral heterogeneity has also been demonstrated through RNA sequencing of three spatially distinct regions from the same MPNST. Sixteen differentially expressed genes were identified among the three regions (such as *EEF1A1*, *RPS27*, *RPLP1*, *ATP5A1*, *HSPB1*, and *H3C3*) involved in protein synthesis and translation, energy metabolism, stress response, and epigenetic regulation [62,63]. In addition, distinct mutational landscapes were also observed, including missense, frameshift, and synonymous variants, as well as unique CNVs, indicating the coexistence of multiple tumor subclones. Notably, one of the three regions harbored a frameshift variant in *CSK* gene, encoding a C-terminal Src kinase that has previously been found to act as a tumor suppressor [63]. Alterations in *CSK* may provide another mechanism for disrupting the PRC2 complex in MPNSTs, similar to findings in breast cancer where *CSK* loss alters the expression of PRC2 subunits *EZH2* and *SUZ12* [64].

A recent study [65] exhibited a clear association between genomic and transcriptomic alterations, with copy number gains leading to increased activity of proliferative pathways and defining a transcriptomic subtype associated with poor prognosis. MPNSTs were stratified into two main transcriptomic subtypes. The “immune-active” subtype, which comprised approximately 44% of tumors, exhibited strong immune signaling, was more frequently low-grade, and correlated with a favorable prognosis. In contrast, the “immune-deficient” subtype, accounting for about 56% of tumors, was associated with aggressive disease, high proliferative activity, complex genomic alterations, PRC2 loss, and *TP53* aberrations. The immune-deficient subtype showed greater genomic instability, with a significantly higher burden of CNVs (notably 8q gains associated with PRC2 loss) and LOH compared with immune-active tumors. These tumors were often triploid, reflecting increased genomic complexity.

Approximately 29% of genes upregulated in immune-deficient tumors showed concurrent copy number gains, mainly affecting proliferative signaling pathways regulated by *E2F*, *MYC*, and the G2/M checkpoint, as well as oncogenes such as *EGFR*, *SMO*, *XPO1*, and *EZH2*. Similarly, about 20% of downregulated genes exhibited concurrent copy number losses, including key tumor suppressors such as *PTEN*, *RB1*, *JAK1*, *JAK2*, *CYLD*, *CBL*, *SDHB*, and *SDHD* [65]. In the same study, tumors with H3K27me3 loss were more aggressive, exhibiting a higher genomic mutation burden and lower immune activity. Instead, those with retained H3K27me3 exhibited stronger immune cell infiltration, suggesting a more favorable immune response and better clinical prognosis [9].

Moreover, numerous dysregulated long non-coding RNAs (lncRNAs) and microRNAs (miRNAs) have been identified in NF1-associated malignancies and are recognized as potential tumor biomarkers. miRNAs, which can act as either tumor suppressors or oncogenes (oncomiRs) in diverse human cancers, play a significant role in NF1 pathogenesis. Notably, miR-486-3p and miR-193a-5p, which target *PTEN* and *KRAS*, respectively, are upregulated in PN compared to dermal neurofibromas. MiR-486-3p may serve as a predictive marker for NF1 progression or as a discriminative marker between plexiform and dermal neurofibroma patients [66].

Moreover, upregulation of hsa-miR-100-5p, hsa-miR-16-2-3p, hsa-miR-4508, and hsa-miR-885-5p, along with downregulation of hsa-miR-107 and hsa-miR-4433b-5p, represent a specific NF1 signature. These six miRNAs regulate multiple genes involved in key cancer-related pathways such as ERK/MAPK and PI3K/AKT/mTOR, the main downstream pathways of neurofibromin [67].

Among lncRNAs implicated in NF1, *ANRIL* is the most studied. Located at chromosome 9p21.3, it plays a role in PN biology. The specific variant rs2151280-T has been associated with increased PN burden, reduced *ANRIL* expression, and a potential correlation with milder NF1 symptoms and optic glioma development. Another lncRNA, *H19* (11p15.5), has oncogenic roles in various cancers. It functions as a molecular sponge for miR-107, thereby regulating *NF1* expression and promoting tumor development [66].

Most neoplastic cells in MPNST lose Schwann cell markers, indicating dedifferentiation to a more primitive state. These cells also overexpress factors associated with early neural crest specification, including *TWIST1*, *SOX9*, *SNAI2*, *OTX2*, *PAX3*, and *PAX6*, which highlights developmental reprogramming during malignant transformation. Pseudotemporal trajectory analysis of tumor cells demonstrates a continuum from atypical neurofibroma (with a Schwann cell identity) to MPNST (with a neural crest-like cell identity), further supporting the hypothesis that these tumors follow developmental trajectories of the neural crest lineage [60].

### 3.5. Epigenomic Profiling in ANNUBP-to-MPNST Transition

The first genome-wide methylome profiling study, using pooled cohorts (10 MPNST, 10 neurofibromas, 6 normal Schwann cells), found no global hypomethylation in MPNST [68]. Instead, it identified focal, cancer-associated differentially methylated regions (DMRs) with distinctive repeat-element biases, such as hypomethylation at satellite (ARLα, SATR1) and SINE repeats, hypermethylation at LINEs, and strong enrichment of hypermethylated DMRs in CpG island (CGI) shores. Integration of methylation data with external expression datasets showed that genes linked to CGI-shore DMRs separate benign from malignant phenotypes, highlighting gene-level concordance, such as *SOX10* and *CDKN2A* promoter hypermethylation, with their reduced expression. Together, these findings support selective, functionally relevant epigenomic reprogramming in the Schwann-cell lineage, rather than a global methylation shift. They also suggest that repeat-element hypomethylation, especially at specific satellite subtypes, may occur early in the benign-to-malignant continuum [68].

Building on the genome-wide DNA methylation map defined by Feber et al. [68] which delineated focal and expression-linked DMRs rather than global hypomethylation, histone-modification profiling has revealed a complementary axis of epigenetic change, centered on PRC2 loss and H3K27me3 depletion. As noted previously, a critical step in the transition toward malignancy is disruption of the PRC2, most often through LOF mutations or deletions in *SUZ12*, *EED* and *EZH2*. This disruption marks a pivotal point in the progression from ANNUBP to MPNST. Rather than enforcing global chromatin repression, these alterations reshape the enhancer landscape and reprogram Schwann-cell differentiation states [26]. Functionally, PRC2 loss promotes a dedifferentiated, neural-crest-like phenotype associated with increased invasiveness and therapy resistance. From a diagnostic standpoint, the resulting loss of H3K27me3 immunostaining remains a practical marker of PRC2 deficiency, although its sensitivity and specificity are not absolute [25,69,70].

More recently, integrative methylation and transcriptomic profiling by Suppiah et al. [60] refined the MPNST landscape into two stable epigenetic subclasses, linked to distinct cell-state programs and signaling dependencies. This work bridges methylation-defined and transcriptional trajectories, emphasizing how PRC2-dependent and PRC2-independent chromatin remodeling jointly shape malignant progression and therapeutic response.

Although widely used, H3K27me3 immunohistochemistry may also exhibit reduced staining in fibrosarcomatous dermatofibrosarcoma protuberans, limiting its standalone specificity [25]. Integrating IHC results with methylation profiling or targeted sequencing improves diagnostic accuracy in such challenging cases [70].

Complementing this methylome-driven taxonomy, recent multi-omics maps comprising precursor lesions (e.g., ANNUBP) show that MPNSTs segregate into two biological trajectories defined by H3K27me3 status. PRC2-deficient/H3K27me3-loss cases undergo broad chromosomal losses that can culminate in near-haploidization, followed by whole-genome doubling with recurrent 8q amplification, and display an immune-depleted microenvironment. In contrast, H3K27me3-retained tumors follow alternative, non-haploid trajectories and are comparatively immune-enriched. Together, these profiling layers help position ANNUBP along the NF1 tumor continuum and provide a practical framework for monitoring progression [9].

At the DNA-methylation level, integrative analyses further link PRC2/H3K27me3 loss to a shift toward hypermethylation at defined CpG sets. This identifies significant signaling pathways, most prominently within the IL17 axis (e.g., *IL17D*/*IL17RD*), that correlate with progression, metastasis, and with H3K27me3 status across independent MPNST cohorts. These tissue-based signatures help discriminate benign from transformed lesions in NF1 and provide candidates for biomarker development [71].

Interestingly, integrated transcriptomic and epigenomic analyses revealed two distinct molecular MPNST subgroups with different biological characteristics and outcomes. MPNST-G1 is PRC2-deficient, exhibits CpG-island promoter hypermethylation and activation of the Sonic Hedgehog (SHH) pathway, which support tumor progression resulting in a worse prognosis. In contrast, MPNST-G2 is PRC2-intact and shows global hypomethylation and activation of the WNT/β-catenin/Cyclin-D1 signaling pathway, a specific hallmark of this subtype [60]. Mechanistically, WNT activation, driven, for instance, by the knockout of its negative regulator APC, drives malignant behavior in human Schwann cell models and sustains MPNST growth [72,73].

Additional networks implicated in MPNST pathogenesis include HIPPO/YAP-TAZ signaling pathway, whose activation has been documented in patient cohorts and functional models [72,73,74].

Moreover, methylation changes in key regulatory loci directly influence distinctive transcriptional programs. For instance, promoter hypermethylation of *SOX10* and *CDKN2A* correlates with their reduced expression, supporting their role as functional mediators of lineage reprogramming and cell-cycle escape [68].

Furthermore, epigenetic alterations and differential expressions have been observed in other genes, such as those in the IL17 pathway, where differentially methylated CpG sites have been associated with MPNST progression [71]. The *RASSF1A* gene has also been discussed in the study by Danielsen et al. which reported promoter hypermethylation in 60% of the analyzed samples. This epigenetic silencing correlates with an unfavorable prognosis in patients with NF1-associated MPNST, suggesting that *RASSF1A* may represent a potential prognostic marker [75].

### 3.6. Integrative Overview of Multi-Omics Findings

The multi-layered omics analyses presented in this section collectively outline a coherent molecular trajectory from PN to ANNUBP and, ultimately, to MPNST.

Despite heterogeneity across individual studies, several convergent principles emerge.

First, genomics remains the backbone of NF1-associated tumor evolution. Biallelic *NF1* loss serves as the initiating event, followed by early *CDKN2A/B* deletion, which defines ANNUBP. Subsequent widespread chromosomal instability and PRC2 inactivation culminate in MPNST. Together, these events drive the transition of Schwann cells from a proliferative but relatively stable state to a highly aneuploid, epigenetically deregulated, and malignant phenotype.

Second, transcriptomics and spatial omics data demonstrate that malignant progression is driven not only by genetic alterations but also by the rewiring of lineage identity, hypoxia-induced immune evasion, and remodeling of the tumor microenvironment. Immune-active versus immune-deficient transcriptomic subtypes, as along with PRC2-dependent versus PRC2-independent regulatory programs, represent two biologically meaningful and prognostically significant classifications.

Third, epigenomic profiling provides another dimension, revealing distinct DNA methylation classes and H3K27me3-defined epigenetic trajectories.

Integration of methylation and expression data highlights key regulators, such as *SOX10*, *CDKN2A*, and genes in IL17-axis, whose altered chromatin states accompany lineage dedifferentiation, senescence escape, and metastatic behavior. PRC2 loss emerges as a central event that links genomic instability, epigenetic deregulation, and transcriptomic reprogramming.

Together, these multi-omics approaches offer a unified model of the stepwise, yet heterogeneous, evolution of NF1-associated tumors, a process shaped by both genetic mutations and environmental pressures. Table 1 provides an integrated overview of the major multi-omics studies discussed above, summarizing patient cohorts, technologies used, recurrently altered genes and pathways, and their diagnostic or prognostic relevance. Each study includes details on the analyzed patient cohort, reported in the cited references (NF1-associated or sporadic PN, ANNUBP, MPNST), the omics technology employed, the main findings encompassing identified mutations (e.g., *TP53*, *CDKN2A*, *SUZ12*), biomarkers, and dysregulated molecular pathways, as well as the corresponding clinical implications, including diagnostic and prognostic value and potential therapeutic targets. Abbreviations: ANNUBP, atypical neurofibromatous neoplasm of uncertain biological potential; cfDNA, circulating cell-free DNA; CNV, copy number variation; DNAm, DNA methylation; IHC, immunohistochemistry; MPNST, malignant peripheral nerve sheath tumor; PN, plexiform neurofibroma; PRC2, polycomb repressive complex 2; SNV, single nucleotide variant; ULP-WGS, ultra-low-pass whole-genome sequencing; WES, whole-exome sequencing; WGS, whole-genome sequencing; PNST, peripheral nerve sheath tumor; cNF, cutaneous neurofibroma; ANF, atypical neurofibroma [9,11,26,30,43,58,61,67,76,77,78,79,80,81,82,83,84,85,86].

## 4. Liquid Biopsy: A Novel Approach for Early Detection and Disease Monitoring

### 4.1. Circulating Tumor DNA (ctDNA) and NF1-Specific Mutational Signatures

Circulating cell-free DNA (ccfDNA) was first described in 1948 by Mandel and Metais in both healthy subjects and patients. CcfDNA is normally present in blood at low concentrations (1–10 ng/mL), but its levels increase in various physiological and pathological conditions, including infections, trauma, and tumors. In 1994, tumor-specific DNA fragments, containing mutations identical to those found in tumor tissue (*K-RAS* gene), were identified for the first time in the plasma of patients with pancreatic cancer. This fraction was defined as circulating tumor DNA (ctDNA) [87]. As ctDNA is derived from tumors and released through apoptosis, necrosis, or extracellular vesicle release, researchers typically evaluate potential SNVs, structural variants, or epigenetic alterations using highly sensitive methods such as PCR, targeted NGS, or ultra-low pass whole genome sequencing (ULP-WGS). In addition, a particularly interesting aspect of ctDNA concerns the fragment length evaluation. The distribution of ctDNA fragment sizes constitutes a molecular signature, often defined as fragmentation signature, that can distinguish between different clinical states.

Integrating ctDNA analysis or ccfDNA fragmentomic approaches could be a valuable tool for improving the early diagnosis, risk stratification, and longitudinal monitoring of patients at risk of MPNST transformation [79,80,82]. For instance, patients with MPNST exhibit a higher proportion of shorter fragments compared to those with PN or healthy controls [82]. Recently, Taylor Sundby et al. analyzed plasma-derived fragment distributions and identified an altered fragmentation pattern characteristic of MPNST, which was absent in patients with only PN or ANNUBP.

In addition to fragment-length alterations, another analytical dimension of ctDNA involves studying the CNVs, which reflect the genomic instability characteristic of neoplastic transformation. Several studies [79,82,87] have demonstrated the ability to detect CNVs in cfDNA with high specificity in patients with MPNST, in some cases anticipating the radiological diagnosis of malignant transformation [80]. For example, Szymanski et al. used ULP-WGS at ~0.6× coverage, followed by in silico selection of 90–150 bp fragments, for quantifying the plasma tumor fraction and distinguish patients with MPNST from those with PN. The study achieved an area under the curve (AUC) of 0.83, with 75% sensitivity and 91% specificity at baseline, leading to an overall accuracy of 89% in serial sample analysis [79]. The plasma tumor fraction significantly correlated with tumor burden, as assessed by the standardized quantitative RECIST SLD (Response Evaluation Criteria In Solid Tumors as Sum of the Longest Diameters of the target lesions) method (Pearson correlation coefficient, r = 0.39), as well as with therapeutic response and minimal residual disease (MRD). This suggests that ctDNA changes may anticipate those observable by radiological imaging.

The copy number profile observed in ccfDNA mirrored that of the tumor, showing large chromosomal gains (1q, 7p, 8q, 9q, and 17q) and losses (6p and 9p), as well as focal deletions in the *CDKN2A*, *SUZ12*, and *SMARCA2* genes associated with the transition from PN to ANNUBP. Additionally, *NF1* loss was detected in the plasma of patients with PN and MPNST but not in healthy controls, confirming the tumor origin of ccfDNA and its potential as a non-invasive biomarker of progression [79].

Beyond the analysis of individual CNVs, a more comprehensive approach based on Genome-wide Aneuploidy Scoring (GAS) has been proposed [87]. GAS measures the overall degree of chromosomal instability in the ccfDNA. Specifically, GAS alone shows limited sensitivity (~33%) in detecting MPNSTs; on the other hand, combining it with focal subchromosomal events (e.g., loss of *TP53* or *SUZ12*) and/or specific mutations in ctDNA increases sensitivity to approximately 50%, while maintaining 97% specificity.

Notably, a distinctive loss of *SUZ12* was observed in the cfDNA derived from a PN patient, which was detected 25 months before the clinical diagnosis of MPNST, highlighting the predictive potential of liquid biopsies for identifying malignant transformation at early stages [80,87]. Another promising application of ctDNA analysis involves the study of epigenetic alterations, particularly DNA methylation patterns and histone modifications, which reflect transcriptional and structural changes associated with neoplastic progression [9]. Sundby et al. showed that combining CNV signals and methylation changes in ctDNA improves diagnostic sensitivity compared to methods based exclusively on genomic variations.

Furthermore, genome-wide methylation analysis by Tomczak et al. identified a panel of 53 specific, differentially methylated CpG sites associated with MPNST. These CpGs distinguish MPNST from PN or ANNUBP. The identified loci map to genes such as *OX9*, *RUNX1*, *PRDM16*, *PTPRN2*, and *COL23A1*, which are involved in transcriptional regulation and tumorigenesis. Therefore, this methylation classifier represents a non-invasive, functional diagnostic tool for early NF1-associated tumor diagnosis and screening [88].

Despite growing evidence supporting the utility of ctDNA in NF1-associated tumors, several limitations complicate its clinical implementation. The rarity of these neoplasms, particularly MPNSTs, combined with the low tumor burden in primary lesions, makes collecting sufficiently large patient cohorts challenging [80]. Furthermore, ctDNA concentrations in patients with NF1 are extremely low. Recent studies have shown only slightly increased ccfDNA levels compared to healthy controls, with ctDNA detectability limited to a few cases [87]. This scenario requires highly sensitive sequencing techniques and optimized bioinformatic pipelines to ensure reproducible results and reduce the risk of false negatives.

Technical variability in ccfDNA isolation, sequencing depth, and data processing remains a major challenge. To date, no plasma-based assay specific for NF1 detection has been clinically validated. Indeed, while several liquid biopsy panels, such as FoundationOne^®^ Liquid CDx (Foundation Medicine, Inc., Cambridge, MA, USA), Guardant360^®^ CDx (Guardant Health, Inc., Redwood City, CA, USA), PGDx elio™ Plasma Focus Dx (Personal Genome Diagnostics, Baltimore, MD, USA) and Shield^®^ (Guardant Health, Inc., Redwood City, CA, USA), have been approved by the FDA for various solid tumors (e.g., lung, prostate and colorectal cancers), there are currently no clinically validated assays for ctDNA in MPNST or other NF1-associated tumors.

As highlighted by Sundby et al. [82] and Rahrmann et al. [88], approaches based on fragmentomics, copy number profiling, and methylation signatures have demonstrated high diagnostic potential. However, their integration into clinical practice requires prospective longitudinal studies in large cohorts to validate their sensitivity, specificity, and prognostic value.

Overall, ctDNA is a promising, noninvasive biomarker for early diagnosis and monitoring of malignant transformation in NF1, with potential to aid in addressing intra-tumor heterogeneity and supporting the search for therapeutic targets. However, further clinical validation and standardized protocols are necessary before ctDNA can be effectively integrated into clinical practice.

### 4.2. Circulating Cell-Free RNA (ccfRNA) and Gene Expression Profiling

Circulating cell-free RNA (ccfRNA) is an emerging analyte of great interest for monitoring various pathological conditions over time, including cancer. Present in plasma, ccfRNA provides complementary information to total RNA from blood cells, as it originates from both viable circulating cells and peripheral tissues via active secretion or apoptotic cell death processes. Due to these characteristics, ccfRNA is becoming a promising liquid biopsy tool capable of reflecting the body’s physiological or pathological state in real time [89].

Alterations in miRNA profiles have been described in numerous pathological conditions, including various cancers, where they can act as oncogenes or tumor suppressors, influencing proliferation, apoptosis, and metastasis [90]. Consequently, quantifying miRNAs in plasma has been proposed as a non-invasive method for early cancer diagnosis and risk stratification.

In the context of NF1, available data on circulating microRNAs is limited but promising. One of the few systematic investigations to date was conducted by Napolitano et al. They analyzed a cohort of 126 NF1 patients with different phenotypes stratified into five phenotypic groups (G1–G5, in order of increasing aggressiveness), and compared them with 128 healthy subjects, aiming to outline a potential miRNA profile associated with the disease. They identified 87 differentially expressed miRNAs, selecting 37 primary candidates. Notably, miR-16-2-3p, miR-100-5p, miR-4508, and miR-885-5p were significantly overexpressed, while miR-107 and miR-4433b-5p were downregulated in NF1 patients compared to controls, suggesting a potential molecular signature of the disease. Although no significant differences were observed among NF1 phenotypic subgroups, but Ingenuity pathway analysis revealed a strong correlation between candidate miRNAs and pathways crucial in NF1-related tumorigenesis, including RAS/ERK/MAPK and PI3K/AKT/mTOR. Given that many of these miRNAs are already deregulated in other RAS-dependent cancers, their functional validation in NF1 could contribute to defining novel diagnostic or prognostic biomarkers and, potential new therapeutic targets [67].

In addition to miRNAs, several circulating coding RNA biomarkers have been proposed as potential indicators of malignant transformation in NF1 patients. Together with ccfRNA and ctDNA data, these biomarkers could represent reliable tools for longitudinal monitoring. For example, adrenomedullin levels are increased in patients with MPNST, suggesting a role in tumor angiogenesis. sAXL and melanoma inhibitory activity (MIA) correlate with tumor size and burden, while IGFBP-1 and RANTES are significantly overexpressed in MPNST compared to PN. Additionally, elevated levels of the pro-inflammatory cytokines IL-6, TNF-α, and IFN-γ indicate a state of systemic inflammation associated with tumor progression [87].

Further therapeutic targets in NF1-related tumors have been identified through gene expression profiling and phosphoproteomic screening studies. In particular, Aurora kinase A (*AURKA*) expression is 7.9-fold higher in human and murine MPNST samples than in healthy, and its pharmacological inhibition reduces tumor cell survival in vitro and in vivo [23]. Similarly, Polo-like kinase 1 (*PLK1*) has been identified as a crucial driver of proliferation in MPNST and Schwannoma cell lines. Like AURKA, PLK1acts at the G2/M transition of the cell cycle. PLK1 inhibitors such as BI6727 have been shown to stabilize tumor volume in MPNST xenografts, suggesting a potential therapeutic role, albeit within a limited therapeutic window [91].

Finally, the loss of the epigenetic marker H3K27me3, characteristic of MPNSTs as previously discussed, can result not only from genetic alterations or alternative epigenetic mechanisms affecting the PRC2 complex but also from microRNA-mediated regulation or *EZHIP* overexpression, as observed in other tumor types such as Merkel cell carcinoma [44].

These observations underscore the importance of integrating epigenetic, transcriptional, and proteomics data when studying NF1-associated tumor progression. Combining cfRNA and ctDNA analysis from the same sample increases diagnostic sensitivity by capturing both transcriptomic information and genomic instability.

## 5. Genomic Data Consortia in NF1: Collaborative Efforts in Data Sharing and Research Advancement

The rapid evolution of omics technologies and the development of high-throughput platforms have allowed high resolution study of genetic disorders such as NF1-associated disease. Advances in sequencing, including WES/WGS, RNA profiling and methylation analysis, have enabled deeper, multi-layered characterization of these disorders, consequently generating datasets, even for poorly characterized conditions like ANNUBP and MPNST. In the big-data era, however, the focus has moved from data generation to data sharing and analysis; this translated into the need for centralized and harmonized platforms.

Several collaborative initiatives have been proposed, aiming at collecting data, gathering resources, and sharing efforts and knowledge to advance our understanding of these conditions. Among the most widely used and data-richest platforms for NF1-related research are the Neurofibromatosis Therapeutic Acceleration Project (NTAP) [92], the Children’s Tumor Foundation (CTF) Synodos [93], and the National Cancer Institute—Genomic Data Commons (NCI-GDC) [94]. NTAP is a collaborative action focused on leading and funding research in NF1-related plexiform and cutaneous neurofibromas. It aims to accelerate therapies development through a collaborative and transparent approach, covering multiple initiatives such as cell and tissue biobanks, animal and cell model systems, omics data generation and collection, and therapeutic screening. All data are accessible via the NF Data Portal (available online: https://nfdataportal.org/, accessed on 27 November 2025), an open repository with controlled access for NF1-related studies [95].

To further advance in NF-related research, NTAP has partnered with organizations such as the Children’s Tumor Foundation CTF, a non-profit medical foundation dedicated to drug discovery for NF1 and Schwannomatosis. Recently, CTF launched CTF Synodos [96], a collaborative NF-centered consortium designed to accelerate discovery by sharing unpublished and curated data, knowledge, and workflows across participating institutions and research groups. Data openness and transparency are reached through online platforms, such as Synapse [95] available online: https://nf.synapse.org/ (accessed on 27 November 2025).

The third major resource is represented by NCI-GDC (available online: https://gdc.cancer.gov/, accessed on 27 November 2025), the genomic division of the NIH’s Cancer Institute. NCI-GDC provides a large repository of multi-omics data, curated metadata, harmonized analysis pipelines, integrated computational tools, and interconnections with other platforms such as The Cancer Genome Atlas (TCGA) [97]. Although not exclusively focused on NF-related conditions, NCI-GDC comprises NF1 and MPNST samples along with molecular and omics profiles, offering valuable opportunities to advance NF1-related research.

In addition, the international GeM Consortium provides multi-omics datasets focused on MPNST evolution, comprising multi-regional WES and WGS, RNAseq, DNA methylation, and cfDNA data [9]. A summary of these resources is reported in Table 2.

A relevant contribution to data sharing and integration for peripheral nerve tumors associated with NF1 is represented by the recently updated Johns Hopkins NF1 Biorepository [99]. This computational resource collects and harmonizes omics data (genomics, transcriptomics, and epigenomics) along with curated clinical information from numerous samples of PN, ANNUBP, and MPNST. This project serves as a centralized platform for translational research, promoting comparative and integrative multi-omics analysis, and supporting the definition of “evolutionary trajectories” that lead from benign lesions to malignant tumors.

Together, these efforts illustrate how strategic collaborative partnerships may accelerate disease characterization, empower basic research, and enable biomarkers discovery, mechanistic modelling, and variants prioritization that can be directly translated into personalized medical approaches. As omics technologies continue to advance, the values of such initiatives will depend not only on data generation but, crucially, on the creation of transparent, interoperable and curated repositories.

## 6. Conclusions and Future Perspectives

Research into the molecular and cellular mechanisms of MPNST pathogenesis in NF1 has been markedly refined through multi-omic analyses encompassing genomic, transcriptomic, and epigenomic profiling. Beyond *NF1* and *CDKN2A* inactivation, loss of PRC2 function and alterations in key signaling pathways such as RAS/MAPK, PI3K/AKT/mTOR, and Wnt/SHH, are now recognized as central drivers of tumor initiation and progression.

A multistep evolutionary trajectory from PN to ANNUBP and, ultimately, to MPNST has been proposed. Comprehensive multi-omic mapping has revealed two distinct evolutionary trajectories of MPNSTs distinguished by H3K27me3 status and PRC2 dependency. PRC2-deficient (H3K27me3-loss) tumors exhibit widespread chromosomal losses, near-haploidization followed by whole-genome doubling, recurrent 8q amplification, and an immune-depleted microenvironment. In contrast, PRC2-intact (H3K27me3-retained) tumors show non-haploid karyotypes and greater immune infiltration [9].

Moreover, transcriptomic and methylation-based subtyping have delineated biologically distinct MPNST classes, such as immune-active versus PRC2-deficient/proliferative subtypes, and SHH- versus WNT-driven mechanisms, which correlate with prognosis and therapeutic vulnerabilities.

Despite advances from genomic studies in identifying key events driving neurofibroma progression, major challenges remain in predicting malignant transformation potential. The rarity and heterogeneity of MPNST hinder biomarker validation and limit the power of therapeutic trials.

In parallel, liquid biopsy approaches based on cfDNA and cfRNA are emerging as powerful non-invasive tools for detection, monitoring and molecular stratification of NF1-associated tumors. However, greater standardization and validation in multicenter cohorts are urgently needed.

Future research should be focused on AI-based integration of multi-layered omics, spatial, transcriptomics, and single-cell analyses to more accurately map the tumor ecosystem, identify actionable dependencies, and track clonal evolution in real time. Such advances hold the potential to transform NF1 management and surveillance, paving the way toward precision oncology and improved patient outcomes.

## Figures and Tables

**Table 1:** Summary of omics studies in NF1-related tumors (PN–ANNUBP–MPNST continuum).

Patients Cohort	Tested Omics Technology	Key Findings (Genes/Biomarkers/Pathways)	Clinical Implications (Diagnostic/Prognostic)	Ref.
NF1, cutaneous neurofibromas (cNF, benign): 11 patients (40 tumors)	WGS, RNAseq, SNP array	Biallelic *NF1*, low secondary drivers; baseline cNF transcriptome with ECM/immune signatures	_	[76]
NF1, plexiform neurofibroma derived Schwann cell models (PN): 7 donors, 11 cell lines (6 deeply profiled)	Targeted/WES, RNAseq, pharmacologic profiling	*NF1* loss: RAS-pathway activity; drug-response resource	Preclinical PN Schwann-cell panel for pharmacogenomic screening; supports testing MEK (selumetinib), mTOR, PI3K, RTK (EGFR/PDGFR), BET, and CDK4/6 inhibitors and combinations	[77]
ANNUBP/atypical neurofibromas (human): 16 ANF (WES); 26 ANF (CNV meta); 5 ANF (RNA-seq); comparators MPNST: 3 (WES), 28 (CNV), 5 (RNA-seq	WES, targeted seq, CNV, RNA-seq	Frequent *CDKN2A* and *SMARCA2* loss; PRC2 intact in ANNUBP; RAS/MAPK feature	_	[30]
ANNUBP vs. cNF/PN vs. MPNST: 40 ANNUBP	DNA methylation (EPIC array)	Distinct methylation class: ANNUBP clusters near benign PN; H3K27me3 largely retained	Diagnostic: methylation profiling aids classification/risk along cNF/PN, ANNUBP, MPNST	[78]
NF1-associated PNST spectrum (cNF/PN, ANNUBP, MPNST): 35 tumors	Spatial transcriptomics; RNA-seq	Early immuno-oncogenic programs at ANNUBP; progressive RAS/MAPK and microenvironment remodeling	_	[61]
MPNST (NF1-associated, sporadic, post-radiation): NF1-associated 27; sporadic 13; post-RT 9	WGS, WES, targeted seq, CNV; PRC2/IHC	Recurrent PRC2 loss (*EED, SUZ12*); frequent *NF1*, *TP53*, *CDKN2A*; RAS–MAPK activation	Diagnostic: H3K27me3 loss by IHC marks PRC2-deficient MPNST; Therapeutic: epigenetic vulnerabilities highlighted	[26]
MPNST 12	WES, SNP array	Recurrent *NF1*, *SUZ12/EED*, *TP53*, *CDKN2A*; broad CNV burden	_	[58]
NF1-associated and sporadic MPNST (GeM Consortium): 95 samples from 90 tumors (61 NF1-related/29 sporadic)	WGS, multi-regional WES, RNA-seq, DNAm, cfDNA	Evolutionary trajectories; CNV-driven progression; convergence on RAS/MAPK and PRC2-loss; cfDNA mirrors tumor alterations	_	[9]
NF1 PN vs. MPNST (liquid biopsy): 16 healthy, 23 PN, 14 MPNST	cfDNA ULP-WGS (fragmentomics and genome-wide aneuploidy profiling)	Plasma fragmentomics and genome-wide aneuploidy profiling distinguish MPNST from pNF and detect malignant transformation	Diagnostic/Prognostic: non-invasive classifier; enables longitudinal surveillance	[79]
883 healthy controls and 7 cNF, 9 PN, 12 MPNST	cfDNA genome-wide aneuploidy profiling and targeted mutation detection	cfDNA detects CNVs and tumor mutations associated with MPNST	Diagnostic: complementary assay for malignant transformation detection	[80]
MPNST (historical aCGH): 24 MPNST and 3 neurofibromas	aCGH (32K BAC)	Broad chromosomal gains/losses (incl. *CDKN2A* locus)	_	[81]
MPNST (n = 35; 15 treated and 10 on treatment), ANF (n = 17), PN (n = 69), and healthy controls (n = 21); 167 cfDNA libraries analyzed	cfDNA fragmentomics	cfDNA fragmentation profiling distinguishes PN–ANNUBP–MPNST; shorter fragments and altered ratios in MPNST; limited CNVs in ANNUBP vs. widespread losses (*SUZ12*, *SMARCA2*, *CDKN2A*) and gains (1q, 7p, 8q, 9q, 17q) in MPNST.	cfDNA fragmentomics enables non-invasive detection and monitoring of NF1 tumor progression.	[82]
42 tumors (37 patients): ANF (n = 5), MPNST (n = 27); 10 tumors: MPNST-like	DNA methylation (EPIC array), RNA-seq, Archer FusionPlex custom panel, Archer VariantPlex custom panel, and Illumina TruSight Oncology 500 panel (TSO500), CNV analysis	Distinct cfDNA fragmentation patterns across PN–ANNUBP–MPNST; shorter fragments and altered short/long ratios in MPNST; limited CNVs in ANNUBP, but extensive CNVs in MPNST (losses: *SUZ12*, *SMARCA2*, *CDKN2A*; gains: 1q, 7p, 8q, 9q, 17q).	DNAm profiling refines MPNST diagnosis, distinguishing low-grade TRK/EGFR-driven subtypes from high-grade PRC2/*CDKN2A*-deficient tumors, linked to aggressive behavior and poorer survival (*p* = 0.0024).	[83]
126 NF1 patients (G1–G5, mild to severe phenotypes) and 128 healthy controls	Small non-coding RNA-seq	Six-miRNA plasma signature identified (↑ miR-16-2-3p, miR-100-5p, miR-4508, miR-885-5p; ↓ miR-107, miR-4433b-5p), linked to RAS/MAPK, PI3K/AKT/mTOR, *PTEN*, *TP53*, and *NF1* pathways.	First serum miRNA panel distinguishing NF1 patients from controls; promising non-invasive biomarker for diagnosis and disease monitoring.	[67]
59 tumors from 55 NF1 patients (35 MPNSTs, 16 plexiform, 8 dermal neurofibromas	Targeted seq, aCGH	Recurrent deletions (*NF1*, *CDKN2A*, *TP53*, *RB1*) and amplifications (*MET*, *HGF*, *PDGFRA*, *EGFR*); co-amplification of *HGF/MET–PDGFRA* indicates activation of p70S6K/mTOR signaling.	Core CNV signature differentiating MPNSTs from benign NF1 tumors; highlights HGF/MET/PDGFRA–mTOR axis as therapeutic target; supports array-CGH for CNV-based diagnosis.	[84]
34 MPNSTs from 27 NF1 patients	*NF1* mutation screening used dHPLC, LOH, MLPA, array-CGH, and sequencing	Frequent *NF1* and *TP53* alterations; 7 novel *NF1* and 4 novel *TP53* mutations; combined *NF1–TP53* loss linked to higher tumor grade and aggressive MPNSTs.	*NF1*–*TP53* co-alterations confirm biallelic NF1 inactivation and cooperative role in MPNST progression; large deletions correlate with higher grade; MLPA/array-CGH improve detection and risk stratification.	[11]
88 MPNSTs (26 NF1-related, 62 sporadic) and 16 benign PN	Targeted seq	*TP53* mutations found in 24% of MPNSTs (mainly missense in exons 5–8); no mutations in benign PN. High p53 protein expression correlated with mutation status (*p* = 0.002). Mutations not enriched in NF1-related vs. sporadic MPNSTs	Demonstrated that *TP53* alterations are relatively rare in MPNSTs and not associated with NF1 status, indicating *TP53* plays a minor role in human tumorigenesis compared to mouse models.	[85]
50 MPNST, 11 NF	WGS, WES, Targeted seq	Identified recurrent *SUZ12* (26%) and *EED* loss-of-function mutations, mutually exclusive with other chromatin remodelers; correlated with H3K27me3 loss. Proposed the *NF1–SUZ12* “three-hit” model for malignant transformation	Established PRC2 loss as a hallmark of MPNST and H3K27me3 loss as a diagnostic and prognostic biomarker. Supported targeting epigenetic regulators in therapy.	[43]
8 MPNST, 1 pNF, 7 cNF	Exome seq, aCGH	Frequent biallelic loss of *SUZ12/EED*, *NF1* inactivation, and *CDKN2A* deletions; rare *TP53* mutations; novel *KDM2B* variant.	Confirmed PRC2 loss as MPNST hallmark and suggested KDM2B as a new chromatin regulator candidate.	[86]

Arrows indicate the direction of differential expression in the plasma miRNA signature: the upward arrow (↑) denotes miRNAs upregulated in NF1 patients compared with healthy controls, whereas the downward arrow (↓) denotes miRNAs downregulated in NF1 patients (small non-coding RNA-seq).

**Table 2:** Overview of the major genomic consortia for NF1, ANNUBP, and MPNST research. The table shows the main collaborative consortia, displaying their name, content, available data type, access model, and key publications (PubMed ID). It summarizes institutional initiatives that generate or provide access to multi-omics data from NF1-associated tumors, including benign neurofibromas, ANNUBP, and MPNST, as well as large-scale repositories that integrate these datasets. bRNAseq: bulk RNA sequencing; cfDNA: cell free DNA; CTF: Children’s Tumor Foundation; DNAm: DNA methylation; GeM: Genomics of MPNST; NCI-GDC: National Cancer Institute Genomic Data Commons; NTAP: Neurofibromatosis Therapeutic Acceleration Project; scRNAseq: single cell RNA sequencing; snRNAseq: single nucleus RNA sequencing; WES: Whole Exome Sequencing; WGS: Whole Genome Sequencing.

Consortium	Content	Data Type	Access Model	Key Publications
NTAP	NF1-derived ANNUBP and MPNST tumor samples; matched normal tissues; cell lines and preclinical models supporting translational studies	WES, WGS, RNAseq	Controlled access	[92,98]
CTF Synodos	Collaborative foundation-led program generating genomic and transcriptomic data from benign NF1 neurofibromas (cutaneous and plexiform) and patient-derived Schwann cell models	WES, WGS, snRNAseq	Controlled access (partially open)	[76,77,93]
NCI-GDC	Central NIH repository aggregating TCGA, TARGET, and sarcoma datasets, including NF1-associated tumors; harmonized data accessible through the GDC portal	WES, WGS, DNAm array, scRNAseq	Open access	[94]
GeM	International consortium investigating MPNSTs; integrates genomic, transcriptomic, methylation, and cfDNA analyses to define tumor evolution and therapeutic vulnerabilities	Multi-regional WES, WGS, RNAseq, DNAm, cfDNA	Controlled access	[9]

## Data Availability

All relevant data are available from the corresponding author upon request.
